# Times change: the NVMO journal going from Dutch to English and from paper to electronics

**DOI:** 10.1007/s40037-014-0130-3

**Published:** 2014-06-17

**Authors:** Janke Cohen-Schotanus

**Affiliations:** Center for Innovation and Research in Medical Education, Groningen, the Netherlands

President NVMO

Times change. The Dutch Association for Medical Education (NVMO) has existed for over 40 years. During almost all this time, the NVMO has had its own journal. In the early years, most articles in the journal focused on best practices and creative ways of teaching. Examples of topics were: the weekly exercise in pharmacotherapy, anatomy in vivo, problem-based learning or whether students can give lectures. We also saw policy and quality discussions about clerkships, simulated patients, selection of students, or facts and fables of teacher training. A regular custom was publication of the annual summary report of the Association for Medical Education in Europe conference. In this way, we informed our members about what was going on in medical education outside the Netherlands. After a couple of years we saw more and more research reports in our journal: reliability of oral examination, which problems do students report to the student counsellor, attitudes of medical students, how three different faculties score on the same progress test or the relation between skills training attendance and study progress. By the year 2000, we had around 400 medical education articles in Dutch, on paper.

In the beginning years, a few enthusiastic NVMO members used the duplicator to print our journal, later we had a real publisher. Especially during the last decade the organization of the journal has become more and more professional. A couple of years ago we had the ambition to move from a club journal to a fully-fledged international journal, and Perspectives on Medical Education (PME) was born. PME wants to offer quality papers that are of interest to international readers such as medical teachers, educational developers, policy makers and researchers. We have noticed that authors and readers from all over the world know how to find the journal. The journal is printed five–six times a year.

This issue of the journal is the first not to appear in print. From now on, the issues of PME will only be available electronically. An advantage is that articles are easy to access and published faster. A disadvantage might be that, for the members of the NVMO, the journal is no longer available in print. The days of PME as the club journal are over. Club information can now only be found on our website (www.nvmo.nl).

What is the content of this issue of PME? You will find a literature review about gender and speciality choices during medical education and a commentary on this topic. There are four original articles, commentaries, a summary of Michiel Westerman’s PhD thesis, two short communication papers and a letter. Authors come from the Netherlands, but also from the USA, Canada, UK and Ireland. One article is presented as an eye opener.

The NVMO and its journal have changed over time. If I look back, I see that medical education also changed during the last 40 years. However, there are still a lot of educational practices which have not changed. Why is it so difficult for medical educators to apply research outcomes in practice and to change routines from which we know that they are not effective? In this issue you might find some answers in the article by Cees van der Vleuten: *What would happen to education if we were to take education evidence seriously*? You will find a summary of what we have learned from researching medical education over the last decades.

I sincerely expect that, now and in the coming 40 years, PME will continue to contribute to the quality of medical education by (electronic) publishing research outcomes, commentaries and eye-openers, which can be used in practice.

